# Enhanced Efficiency in Dye-Sensitized Solar Cells by Electron Transport and Light Scattering on Freestanding TiO_2_ Nanotube Arrays

**DOI:** 10.3390/nano7100345

**Published:** 2017-10-24

**Authors:** Won-Yeop Rho, Da Hyun Song, Sang Hun Lee, Bong-Hyun Jun

**Affiliations:** 1 Department of Bioscience and Biotechnology, Konkuk University, Seoul 143-701, Korea; rho7272@gmail.com (W.-Y.R.); shlee.ucb@gmail.com (S.H.L.); 2 Department of Chemistry, Seoul National University, Seoul 151-747, Korea; songssi87@snu.ac.kr

**Keywords:** dye-sensitized solar cell, TiO_2_ nanotube arrays, carbon materials, scattering layer

## Abstract

Dye-sensitized solar cells (DSSCs) were fabricated with closed- or open-ended freestanding TiO_2_ nanotube arrays as photoelectrodes that were decorated with carbon materials and large TiO_2_ nanoparticles (NPs) to enhance energy conversion efficiency. The energy conversion efficiency of DSSCs based on open-ended freestanding TiO_2_ nanotube arrays increased from 4.47% to 5.39%, compared to the DSSCs based on closed-ended freestanding TiO_2_ nanotube arrays. In DSSCs based on the open-ended freestanding TiO_2_ nanotube arrays, the energy conversion efficiency with carbon materials increased from 5.39% to 6.19% due to better electron transport, and that with a scattering layer from 5.39% to 6.24% due to more light harvesting compared to the DSSCs without carbon materials or scattering layer. Moreover, the energy conversion efficiency of DSSCs based on the open-ended freestanding TiO_2_ nanotube arrays with both carbon materials and scattering layer increased from 5.39% to 6.98%, which is an enhancement of 29.50%. In DSSCs based on the TiO_2_ nanotube arrays, the carbon materials can improve electron transport by π-π conjugation, and the large TiO_2_ NPs can enhance the capacity to light-harvest by scattering.

## 1. Introduction

Building-integrated photovoltaics (BIPVs) are one of the essential components in the Smart Grid, and require transparency, flexibility, light weight, low cost, and high power conversion efficiency [1,2]. Since their initial development in 1991 by the Grätzel group [3,4], dye-sensitized solar cells (DSSCs) have been one of the promising BIPV candidates, since their structure is composed of transparent conducting oxide (TCO), an *n*-type nanostructured semiconductor, a visible-light absorber sensitizer, electrolytes (iodide/triiodide, I^−^/I_3_^−^), and a counter electrode [5]. In addition, eco-friendliness and improvement in stability have become one of the foci in recent research into DSSCs. Liquid-state electrolytes consisting of redox couple and a few additives have been used in conventional DSSCs because of their high energy conversion efficiency [3]. However, to improve the stability of the DSSCs, quasi-solid or solid-state electrolytes would be more favored over the liquid-state electrolytes. For the development of eco-friendly devices, water-based DSSCs (i.e., “aqueous DSSCs”) have attracted attention as they exhibit non-flammable, cost-effective, and eco-friendly properties [1,2,5,6,7,8,9].

Mesoporous TiO_2_ nanoparticle (NP) films are generally used in the studies of DSSCs, as the films have a desirable direct band gap (3.2 eV) and a large surface area for adsorbing dyes, both of which help to generate electrons [10,11,12,13]. However, the efficiencies of the films might be limited by their grain boundaries, defects, and innumerous trapping sites that can cause charge recombination and low electron mobility from their structures, which are randomly networked [4,14].

TiO_2_ nanotubes have great potential to overcome the issues of TiO_2_ NP films, since their unique structure enhances electron transport and charge separation by forging direct pathways and by accelerating charge transfer between interfaces [15,16,17]. TiO_2_ nanotube arrays can improve energy conversion efficiencies because of their highly-ordered and vertically-oriented tubular structures and because of their innate advantages. The structure of TiO_2_ nanotube arrays needs to be taken into consideration in order to capitalize on the advantages of TiO_2_ nanotube arrays. Although DSSCs based on TiO_2_ nanotube arrays have a great potential for enhancing power conversion efficiency (PCE), DSSCs based on closed-ended TiO_2_ nanotube arrays—which are the typically employed TiO_2_ nanotube arrays—exhibited lower energy conversion efficiencies than those of DSSCs based on TiO_2_ NP films. Recently, we have demonstrated that open-ended TiO_2_ nanotube arrays in DSSCs, where barrier layers have been removed, exhibited higher PCE [18].

Scattering materials such as TiO_2_, ZrO_2_, and SiO_2_ can improve the energy conversion efficiency by light harvesting. Especially, TiO_2_ is one of the best materials to use for scattering owing to its high chemical stability and dye adsorption capability. As such, TiO_2_ scattering materials have been introduced on mesoporous TiO_2_ NP films for the enhancement of light harvesting [19].

Carbon materials, including carbon nanotubes (CNTs), graphene, or graphite, are promising materials in improving charge separation and electron transport in solar cells due to their enhanced electrical properties by π-π conjugation. The main role of carbon 60 or CNTs in organic solar cells is to function as electron acceptors or charge separators [20]. TiO_2_ composite films with carbon nanotubes or graphene as photoanodes showed better energy conversion efficiency due to the sp^2^ structure of the carbon materials [21,22,23,24]. However, it remains a challenging task to directly incorporate those carbon materials into a well-ordered and vertically oriented tubular structure of TiO_2_ nanotube arrays. 

Herein, we show that large TiO_2_ NPs were introduced onto open- or closed-ended freestanding TiO_2_ nanotube arrays for more light harvesting, and subsequently carbon materials were synthesized into the well-ordered and vertically oriented tubular structure of TiO_2_ nanotube arrays for better electron transport. The performances of DSSCs based on the open- or closed-ended freestanding TiO_2_ nanotube arrays with/without carbon materials and/or large TiO_2_ NPs were compared to elucidate the influence of each component on the energy conversion efficiency of DSSCs.

## 2. Results and Discussion

**Figure 1:**
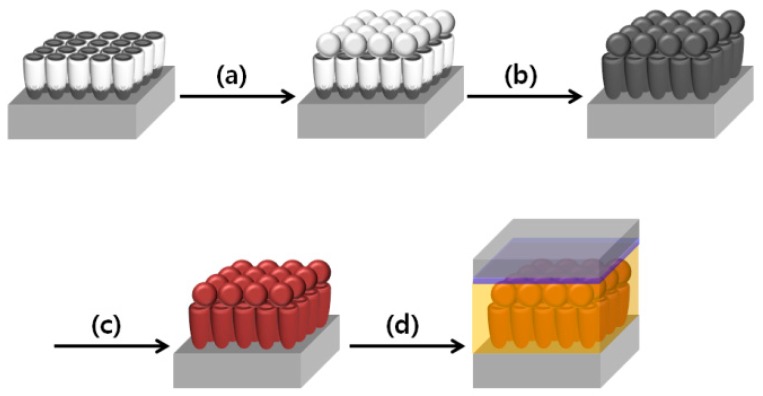
Overall scheme of fabrication of dye-sensitized solar cells (DSSCs) based on closed- or open-ended freestanding TiO_2_ nanotube arrays decorated with large TiO_2_ nanoparticles (NPs) and carbon materials. (**a**) Coating of large TiO_2_ NPs; (**b**) Synthesis of carbon materials by chemical vapor deposition (CVD) method; (**c**) Dye adsorption; and (**d**) Fabrication of the DSSCs.

Figure 1 illustrates the fabrication of DSSCs based on closed- or open-ended freestanding TiO_2_ nanotube arrays with carbon materials and large TiO_2_ NPs as photoanode. The closed- or open-ended freestanding TiO_2_ nanotube arrays were prepared by anodization, and their bottom layer was removed by ion milling process. When the bottom layer is present under the freestanding TiO_2_ nanotube arrays, they are known as “closed-ended” freestanding TiO_2_ nanotube arrays, whereas without the bottom layer, they are called “open-ended” freestanding TiO_2_ nanotube arrays. Both types of freestanding TiO_2_ nanotube arrays were attached on the fluorine-doped tin oxide (FTO) glass, and the large TiO_2_ NPs (~400 nm) were coated onto both types of freestanding TiO_2_ nanotube arrays as shown in Figure 1a. The carbon materials were synthesized by the chemical vapor deposition (CVD) method (Figure 1b), and then the dye (N719) was adsorbed onto both types of freestanding TiO_2_ nanotube array (Figure 1c). Finally, DSSCs were fabricated by assembling the photoanode and counter electrode that were coated with platinum (Pt) on the FTO glass. Electrolyte was injected between the photoanode and counter electrode (Figure 1d).

**Figure 2:**
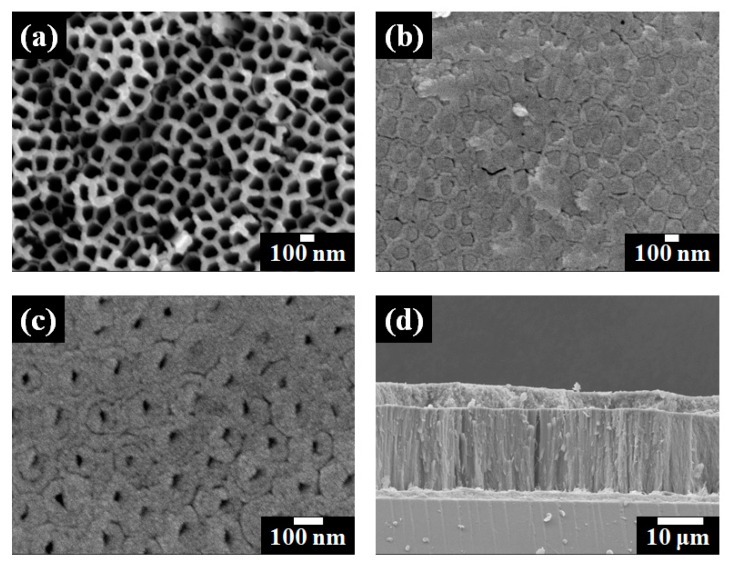
Field emission scanning electron microscope (FE-SEM) images of freestanding TiO_2_ nanotube arrays. (**a**) Top view; (**b**) Bottom view before ion milling process; (**c**) Bottom view after ion milling process of freestanding TiO_2_ nanotube arrays; and (**d**) Side view of freestanding TiO_2_ nanotube arrays and large TiO_2_ NPs on the fluorine-doped tin oxide (FTO) glass.

Figure 2 shows field emission scanning electron microscope (FE-SEM) images of closed- or open-ended freestanding TiO_2_ nanotube arrays. The top view of the freestanding TiO_2_ nanotube arrays can been seen in Figure 2a. The pore size was approximately 100 nm after anodization. Figure 2b shows the bottom view of freestanding TiO_2_ nanotube arrays before the ion milling process. The pattern of the bottom pore size was about 100 nm. Figure 2c shows the bottom view of freestanding TiO_2_ nanotube arrays after the ion milling process, and the pore size was about 30 nm. The size of the bottom pore was much smaller when compared to the sizes of top pore and the pattern of the bottom pore. However, when the levels of thickness were compared, the bottom wall (~35 nm) was much thicker than the top wall of the freestanding TiO_2_ nanotube arrays. In previous works [18,25,26,27,28,29,30], we reported that the shape of TiO_2_ nanotube arrays prepared by anodization were likely to be a corn shape type and that the thicker bottom layer disturbed the electron transport and electrolyte diffusion. Therefore, we suggested that the removal of the bottom layer would facilitate better energy conversion efficiency in DSSCs. Figure 2d shows the side view of freestanding TiO_2_ nanotube arrays and large TiO_2_ NPs on the FTO glass. The thickness of the freestanding TiO_2_ nanotube arrays was approximately 18 μm, and the thickness of large TiO_2_ NPs was approximately 3 μm. 

**Figure 3:**
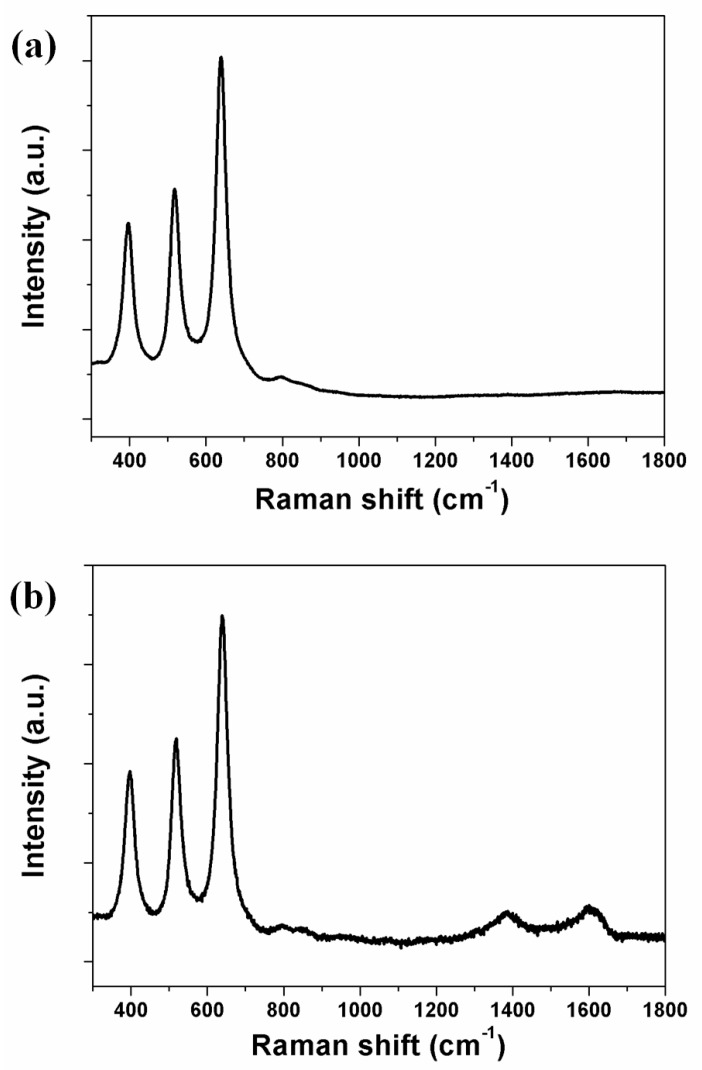
Raman spectra of (**a**) freestanding TiO_2_ nanotube arrays alone and (**b**) freestanding TiO_2_ nanotube arrays with carbon materials.

Carbon materials were synthesized on the freestanding TiO_2_ nanotube arrays by the CVD method, and their structure was confirmed by Raman spectroscopy, as shown in Figure 3. The TiO_2_ nanotube arrays were confirmed at B1g (395 cm^−1^), A1g (517 cm^−1^), and Eg (639 cm^−1^) peaks, indicating that the form of the TiO_2_ nanotube arrays was anatase (Figure 3a). Previously, we have attempted to confirm carbon materials using a transmission electron microscopy (TEM), but experienced difficulties in distinguishing the carbon materials that were located on the wall of TiO_2_ nanotube arrays [30,31]. Using Raman spectroscopy, on the other hand, the carbon materials on the freestanding TiO_2_ nanotube arrays could be confirmed from the G band at 1600 cm^−1^, representing graphite, and the D band at 1384 cm^−1^, representing a disorderly network of sp^2^ and sp^3^ sites in the carbon materials (Figure 3b). In the sp^2^ sites of carbon materials, π-π conjugation had a positive effect on electron transport in enhancing the energy conversion efficiency of DSSCs.

**Figure 4:**
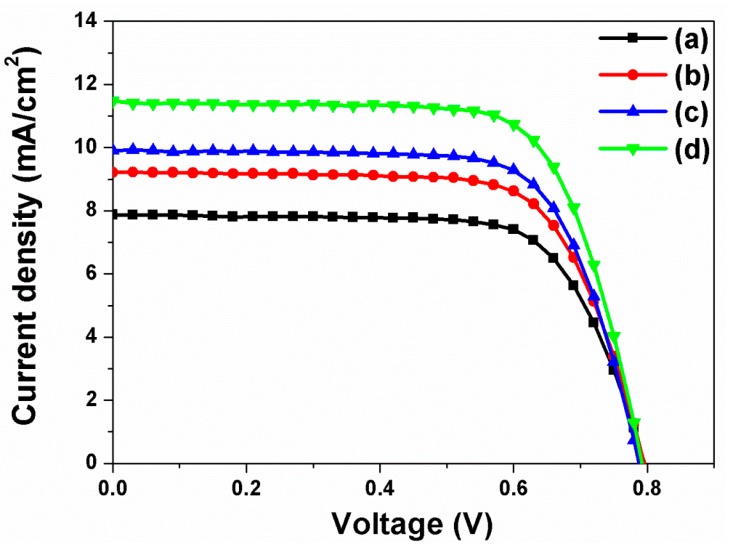
Current density-voltage (*I*-*V*) curves of DSSCs based on closed-ended freestanding TiO_2_ nanotube arrays: (**a**) Without carbon materials and large TiO_2_ NPs; (**b**) With carbon materials; (**c**) With large TiO_2_ NPs; and (**d**) With carbon materials and large TiO_2_ NPs.

The current density-voltage curves (*I*-*V*) of DSSCs based on closed-ended TiO_2_ nanotube arrays with/without carbon materials and/or large TiO_2_ NPs were measured under air-mass (AM) 1.5 sunlight. The results are presented in Figure 4. The open circuit voltage (*V*_oc_), short-circuit current density (*J*_sc_), fill factor (*ff*), and energy conversion efficiency (η) of DSSCs are summarized in Table 1. In DSSCs based on closed-ended TiO_2_ nanotube arrays without carbon materials and large TiO_2_ NPs, the energy conversion efficiency was 4.47%. In DSSCs based on closed-ended TiO_2_ nanotube arrays with carbon materials or with large TiO_2_ NPs, the energy conversion efficiency values were 5.24% and 5.63%, respectively. Although DSSCs based on closed-ended TiO_2_ nanotube arrays with carbon materials had lower dye loading (from 138 nmol/cm^2^ to 124 nmol/cm^2^), as dye could not be adsorbed onto the carbon materials, the energy conversion efficiency values were higher than that of DSSCs without carbon materials and large TiO_2_ NPs. Nevertheless, electron transport would be improved by carbon materials, which can enhance the energy conversion efficiency of DSSCs. In DSSCs with large TiO_2_ NPs, their energy conversion efficiency was higher than that of DSSCs without carbon materials and large TiO_2_ NPs. In this case, their light harvesting would also be improved by large TiO_2_ NPs, which are favorable in enhancing the energy conversion efficiency. Additionally, the DSSCs based on closed-ended TiO_2_ nanotube arrays with carbon materials and large TiO_2_ NPs showed increased energy conversion efficiency from 4.47% to 6.52%, corresponding to a 45.86% enhancement. The results can be attributed to their improved electron transport and light harvesting by π-π conjugation and scattering layer. These results suggest that the increase in energy conversion efficiency of DSSCs depends on the improved ability of electron transport and light harvesting by carbon materials and large TiO_2_ NPs. 

**Figure 5:**
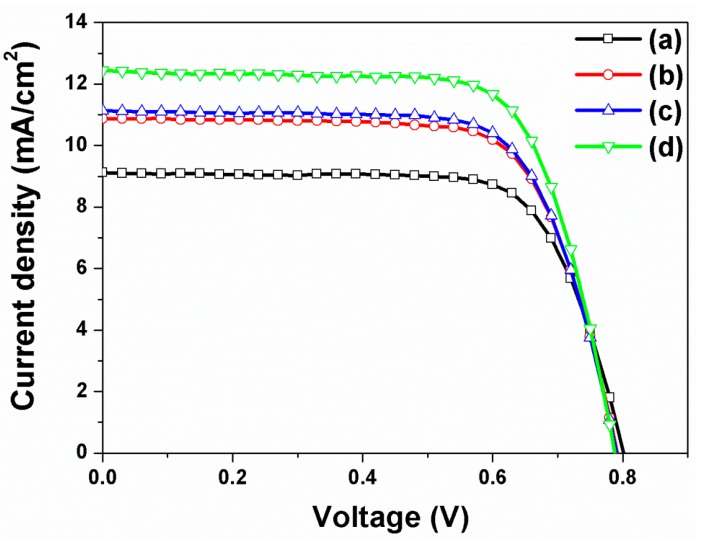
*I*-*V* curves of DSSCs based on open-ended freestanding TiO_2_ nanotube arrays: (**a**) Without carbon materials and large TiO_2_ NPs; (**b**) With carbon materials; (**c**) With large TiO_2_ NPs; and (**d**) With carbon materials and large TiO_2_ NPs.

The current density-voltage curves of DSSCs based on open-ended TiO_2_ nanotube arrays with/without carbon materials were also measured under AM 1.5 sunlight, and the results are presented in Figure 5. The values of *V*_oc_, *J*_sc_, *ff*, and η of DSSCs are summarized in Table 2. In general, the energy conversion efficiencies of DSSCs based on the open-ended TiO_2_ nanotube arrays were higher than those based on the closed-ended TiO_2_ nanotube arrays. Our previous work demonstrated that the electron transfer and electrolyte diffusion of DSSCs based on open-ended TiO_2_ nanotube arrays were better than that based on closed-ended TiO_2_ nanotube arrays [18]. The energy conversion efficiency of DSSCs based on open-ended TiO_2_ nanotube arrays increased from 4.47% to 5.39%. When the carbon materials were decorated on the TiO_2_ nanotube arrays, the energy conversion efficiency of DSSCs based on the open-ended TiO_2_ nanotube arrays increased from 5.39% to 6.19% (14.84% enhancement), which is due to better electron transport by π-π conjugation. When the large TiO_2_ NPs were introduced onto the open-ended TiO_2_ nanotube arrays, the energy conversion efficiency of DSSCs increased from 5.39% to 6.24% (15.77% enhancement), due to more light harvesting by the scattering layer. To capitalize on the synergetic effects between carbon materials and large TiO_2_ NPs in improving energy conversion efficiency, the DSSCs based on open-ended TiO_2_ nanotube arrays were fabricated with carbon materials and large TiO_2_ NPs. The energy conversion efficiency increased from 5.39% to 6.98% (29.50% enhancement). It can be suggested that greater electron transport was facilitated by carbon materials and the better light harvesting by large TiO_2_ NPs, both of which simultaneously improved the energy conversion efficiency of DSSCs. Moreover, the results showed that the energy conversion efficiencies of DSSCs based on open-ended TiO_2_ nanotube arrays were mostly greater than those based on closed-ended TiO_2_ nanotube arrays.

**Figure 6:**
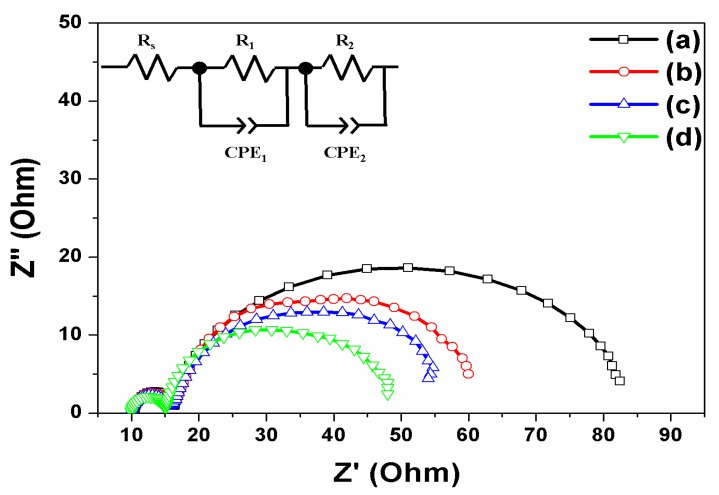
Impedance of DSSCs based on open-ended freestanding TiO_2_ nanotube arrays: (**a**) Without carbon materials and large TiO_2_ NPs; (**b**) With carbon materials; (**c**) With large TiO_2_ NPs; and (**d**) With carbon materials and large TiO_2_ NPs.

The DSSCs based on the open-ended TiO_2_ nanotube array were characterized by electrical impedance spectroscopy (EIS) across the frequency range from 10^−2^ Hz to 10^6^ Hz (as shown in Figure 6), and the fit parameters are listed in Table 3. The applied bias voltage was set at the *V_oc_* with an AC amplitude of 10 mV. The ohmic series resistance (*R_s_*) is a sheet resistance corresponding to the *x*-axis value where a first semicircle begins, as can been seen on the left of Figure 6. When the *R_s_* value in DSSCs based on the open-ended TiO_2_ nanotube arrays is compared, it was similar to that with/without carbon materials and/or large TiO_2_ NPs. The result indicates that the resistance of the sheet against the FTO or the current collector is not affected by the carbon materials and large TiO_2_ NPs. The *R*_1_ value is the sum of the small semicircles at the high frequency. The value was assigned to the parallel combination of resistances and the capacitances at the Pt-FTO/electrolyte and the FTO/TiO_2_ interfaces. The *R*_1_ value of DSSCs without carbon materials and large TiO_2_ NPs was 6.16 Ω, and the *R*_1_ value of DSSCs with carbon materials or large TiO_2_ NPs was 6.23 Ω and 5.91 Ω, respectively. When DSSCs were fabricated with carbon materials and large TiO_2_ NPs, the *R*_1_ value became 5.11 Ω, which was much lower than without carbon materials and large TiO_2_ NPs. The results indicate that a greater amount of electrons were generated by the large TiO_2_ NPs, and that electrons were transferred between the FTO and the TiO_2_. The *R*_2_ value is given by the sum of the large semicircles at low frequency, which is also associated with the resistance and the capacitance at the dye-adsorbed TiO_2_/electrolyte interface and the transport resistance. The *R*_2_ value of DSSCs without carbon materials and large TiO_2_ NPs was 56.27 Ω. When carbon materials were decorated on the TiO_2_ nanotube arrays, the *R*_2_ value decreased to 37.43 Ω, as transport resistance decreased by π-π conjugation. The *R*_2_ value of DSSCs with large TiO_2_ NPs decreased to 34.26 Ω, due to greater electrons being generated by scattering at the dye-adsorbed TiO_2_/electrolyte interface. In DSSCs based on the open-ended TiO_2_ nanotube arrays with carbon materials and large TiO_2_ NPs, the value of *R*_2_ decreased to 29.02 Ω due to the synergistic effect by π-π conjugation and by scattering layer, affecting the FTO/TiO_2_ and TiO_2_/electrolyte interfaces. 

## 3. Materials and Methods 

### 3.1. Materials

Titanium (Ti) plate (99.7% purity, 0.25 mm thickness), ammonium fluoride (NH_4_F, 97.0%), ethylene glycol (99%), hydrogen peroxide (30%), FTO glass, titanium diisopropoxide bis(acetylacetonate) solution (75 wt. % in isopropanol), *n*-butanol, TiO_2_ paste, scattering TiO_2_ paste, titanium chloride (TiCl_4_), dye cis-diisothiocyanato-bis(2,2′-bipyridyl-4,4′-dicarboxylato) ruthenium(II) bis(tetrabutylammonium), N719, chloroplatinic acid hexahydrate (H_2_PtCl_6_·6H_2_O), 1-butyl-3-methyl-imidazolium iodide (BMII), iodine (I_2_), guanidium thiocyanate (GSCN), 4-tertbutylpyridine (TBP), acetonitrile (CH_3_CN), and valeronitrile (CH_3_(CH_2_)_3_CN) were purchased from Alfa Aesar (Haverhill, MA, USA), Showa Chemical Co., (Beijing, China), Daejung Chemical (Shiheung-City, Korea), Pilkington (St. Helens, UK), Aldrich (St. Louis, MO, USA), Solaronix (Aubonne, Switzerland), and Dyesol (Queanbeyan, Australia). 

### 3.2. Preparation of Closed- or Open-Ended Freestanding TiO_2_ Nanotube Arrays

TiO_2_ nanotube arrays were prepared by anodization from a Ti plate that was carried out in an electrolyte composed of 0.8 wt. % NH_4_F and 2 vol. % H_2_O in ethylene glycol. The constant voltage was 60 V DC at 25 °C for 2 h. After the anodization, the Ti plate was annealed at 500 °C for 30 min under ambient conditions to improve the crystallinity of TiO_2_ nanotube arrays. To detach the TiO_2_ nanotube arrays from the Ti plate, a secondary anodization was carried out at a constant voltage of 30 V DC for 10 min and then the Ti plate was immersed in 10% H_2_O_2_ solution for several hours, the results of which are called closed-ended freestanding TiO_2_ nanotube arrays. To prepare open-ended freestanding TiO_2_ nanotube arrays, the bottom of the freestanding TiO_2_ nanotube arrays was removed by ion milling with Ar^+^ bombardment for several minutes. 

### 3.3. Fabrication of DSSCs with Closed- or Open-Ended Freestanding TiO_2_ Nanotube Arrays with Scattering Layer 

The TiO_2_ paste was coated on the FTO glass, and the closed- or open-ended freestanding TiO_2_ nanotube arrays were put on the substrates and then sintered at 500 °C for 1 h under ambient conditions to induce crystallinity and adhesion between the TiO_2_ NPs and freestanding TiO_2_ nanotube arrays. After an annealing step, the ~400 nm TiO_2_ NPs were coated on the freestanding TiO_2_ nanotube arrays for a scattering layer and sintered at 500 °C for 1 h under ambient conditions for their crystallinity. To increase the dye adsorption, the substrates were treated with 0.01 M TiCl_4_ solution for 30 min and sintered at 500 °C for 1 h under ambient conditions for their crystallinity. The substrates were immersed in a dye solution at 50 °C for 8 h, which were then called working electrodes. The working electrodes were sandwiched with a counter electrode that was coated with Pt on the FTO glass by using a 60-μm-thick hot-melt sheet. The electrolyte was filled between the working and the counter electrode. The electrolyte was comprised of 0.7 M 1-butyl-3-methyl-imidazolium iodide (BMII), 0.03 M I_2_, 0.1 M guanidium thiocyanate (GSCN), and 0.5 M 4-tertbutylpyridine (TBP) in a mixture of acetonitrile and valeronitrile (85:15, *v*/*v*). 

### 3.4. Instruments

The morphology, thickness, size, and structure of freestanding TiO_2_ nanotube arrays were confirmed using a FE-SEM (JSM-6330F, JEOL Inc., Tokyo, Japan). The current density-voltage (*J*-*V*) characteristics and the incident photon-to-current conversion efficiency (IPCE) of the DSSCs were measured using an electrometer (Keithley 2400, Keithley Instruments, Inc., Cleveland, OH, USA) under AM 1.5 illumination (100 mW/cm^2^) provided by a solar simulator (1 KW xenon with AM 1.5 filter) or using a McScience (model K3100, McScience Inc., Suwon, Korea) with reference to a calibrated diode.

## 4. Conclusions

We prepared DSSCs based on closed- or open-ended TiO_2_ nanotube arrays as photoanodes that contained the carbon materials and large TiO_2_ NPs to improve energy conversion efficiency. The energy conversion efficiency of DSSCs based on the closed- or open-ended TiO_2_ nanotube arrays with carbon materials had higher energy conversion efficiency than that of DSSCs without carbon materials. This was due to the carbon materials being composed of π-π conjugation on their structure, which is more conducive to electron transports. The energy conversion efficiency of DSSCs based on the closed- or open-ended TiO_2_ nanotube arrays with large TiO_2_ NPs showed greater energy conversion efficiency than that of DSSCs without large TiO_2_ NPs, as large TiO_2_ NPs could generate more electrons by light harvesting. Moreover, the energy conversion efficiency of DSSCs based on the closed- or open-ended TiO_2_ nanotube arrays with carbon materials and with large TiO_2_ NPs showed much higher energy conversion efficiency than that of DSSCs without carbon materials and large TiO_2_ NPs due to their combined effects of enhanced electron transports and electron generation. Our results suggest that the carbon materials and large TiO_2_ NPs could be applied to organic solar cells (e.g., hybrid or perovskite solar cells) to improve their energy conversion efficiency.

## Figures and Tables

**Table 1:** Photovoltaic properties of DSSCs based on closed-ended freestanding TiO_2_ nanotube arrays with/without carbon materials and with/without large TiO_2_ NPs.

Based on Closed-Ended Freestanding TiO_2_ Nanotube Arrays	*J*_sc_ (mA/cm^2^)	*V*_oc_ (V)	*ff*	η (%)	Dye Loading (nmol/cm^2^)
Without carbon materials and large TiO_2_ NPs	7.87	0.80	0.71	4.47	138
With carbon materials	9.22	0.80	0.71	5.24	124
With large TiO_2_ NPs	9.90	0.79	0.72	5.63	149
With carbon materials and large TiO_2_ NPs	11.47	0.79	0.72	6.52	131

Note: *J*_sc_: short-circuit current density; *V*_oc_: open circuit voltage; *ff*: fill factor; η: energy conversion efficiency.

**Table 2:** Photovoltaic properties of DSSCs based on open-ended freestanding TiO_2_ nanotube arrays with/without carbon materials and with/without large TiO_2_ NPs.

Based on Open-Ended Freestanding TiO_2_ Nanotube Arrays	*J*_sc_ (mA/cm^2^)	*V*_oc_ (V)	*ff*	η (%)	Dye Loading (nmol/cm^2^)
Without carbon materials and large TiO_2_ NPs	9.12	0.81	0.73	5.39	150
With carbon materials	10.88	0.79	0.72	6.19	136
With large TiO_2_ NPs	11.14	0.79	0.71	6.24	158
With carbon materials and large TiO_2_ NPs	12.44	0.79	0.71	6.98	141

Note: *J*_sc_: short-circuit current density; *V*_oc_: open circuit voltage; *ff*: fill factor; η: energy conversion efficiency.

**Table 3:** Parameters of impedance spectra of DSSCs based on open-ended freestanding TiO_2_ nanotube arrays with/without carbon materials and with/without large TiO_2_ NPs.

Based on Open-Ended Freestanding TiO_2_ Nanotube Arrays	*R*_s_ (Ω)	*R*_1_ (Ω)	*CPE*_1_ (F)	*R*_2_ (Ω)	*CPE*_2_ (F)
Without carbon materials and large TiO_2_ NPs	10.67	6.16	7.59 × 10^−6^	56.27	1.99 × 10^−3^
With carbon materials	10.43	6.23	8.89 × 10^−6^	37.43	1.94 × 10^−3^
With large TiO_2_ NPs	10.40	5.91	7.91 × 10^−6^	34.26	2.21 × 10^−3^
With carbon materials and large TiO_2_ NPs	10.26	5.11	9.86 × 10^−6^	29.02	2.51 × 10^−3^

Note: *R*_s_: ohmic series resistance; *R*_1_: sum of small semicircles at high frequency; *CPE*_1_*:* constant phase element 1; *R*_2_: sum of large semicircles at low frequency; *CPE*_2_*:* constant phase element 2.

## Supplementary Materials

The following are available online at http://www.mdpi.com/2079-4991/7/10/345/s1.
