# Supplementary material for: Enhanced Efficiency in Dye-Sensitized Solar Cells by Electron Transport and Light Scattering on Freestanding TiO2 Nanotube Arrays
Source: Nanomaterials (Basel). 2017 Oct 24;7(10):345. doi: 10.3390/nano7100345 (PMC5666510; doi:10.3390/nano7100345)
Supplement: Supplementary file 1 [file nanomaterials-07-00345-s001.pdf]

Supplementary Information

Enhanced Efficiency in Dye-Sensitized Solar Cells by Electron Transport and Light Scattering on Freestanding TiO_2 Nanotube Arrays

Won-Yeop Rho [1], Da Hyun Song [2], Sang Hun Lee [1] and Bong-Hyun Jun [1,*]

[1] Department of Bioscience and Biotechnology, Konkuk University, Seoul 143-701, Korea; rho7272@gmail.com (W.-Y.R.); shlee.ucb@gmail.com (S.H.L.)

[2] Department of Chemistry, Seoul National University, Seoul 151-747, Korea; songssi87@snu.ac.kr

* Correspondence: bjun@konkuk.ac.kr; Tel.: +82-2-450-0521

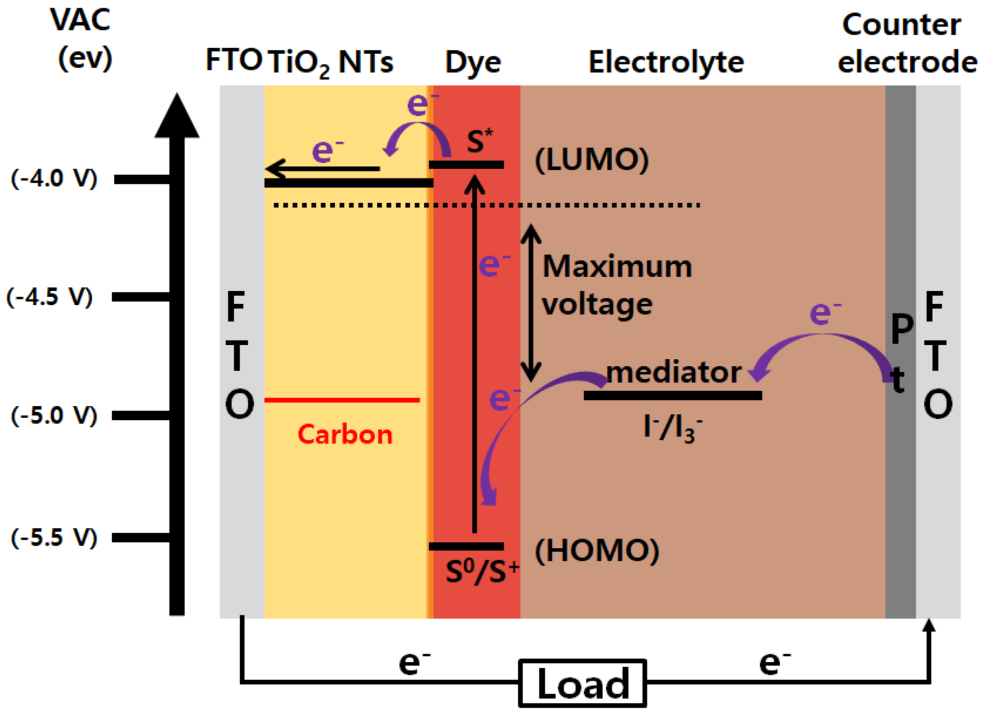

Figure S1. Energy band diagram of DSSCs based on closed- or open-ended TiO_2 nanotube arrays with carbon materials.

When carbon materials were introduced on the closed- or open-ended TiO_2 nanotube arrays, the energy level of the conduction band of the closed- or open-ended TiO_2 nanotube arrays decreased, since work functions of the carbon materials were low (−4.9 eV) as shown in Figure S1. Compared to the DSSCs based on closed-ended TiO_2 nanotube arrays, the V_{oc} value of the DSSCs based on open-ended TiO_2 nanotube arrays showed a small increment (0.80 V to 0.81 V) due to an increase in electron density of the TiO_2 nanotube arrays from higher electron density. However, when carbon materials were introduced on the closed- or open-ended TiO_2 nanotube arrays, the V_{oc} value of the DSSCs showed a slight decrement (0.81 V to 0.79 V) because of the reduced work function of carbon materials. Enhancing electron transport by carbon materials would be helpful to improve the J_{sc} value which is positive to energy conversion efficiency.

Reference

1. Rho, C.; Min, J.-H.; Suh, J.S. Barrier layer effect on the electron transport of the dye-sensitized solar cells based on TiO_2 nanotube arrays. *J. Phys. Chem. C* **2012**, *116*, 7213–7218.
2. Rho, W.-Y.; Chun, M.-H.; Kim, H.-S.; Kim, H.-M.; Suh, J.S.; Jun, B.-H. Ag Nanoparticle—Functionalized Open-Ended Freestanding TiO_2 Nanotube Arrays with a Scattering Layer for Improved Energy Conversion Efficiency in Dye-Sensitized Solar Cells. *Nanomaterials* **2016**, *6*, 117.
3. Rho, W.-Y.; Kim, H.-S.; Kim, H.-M.; Suh, J.S.; Jun, B.-H. Carbon-doped freestanding TiO_2 nanotube arrays in dye-sensitized solar cells. *New J. Chem.* **2017**, *41*, 285–289.
4. Kim, H.-S.; Chun, M.-H.; Suh, J.S.; Jun, B.-H.; Rho, W.-Y. Dual Functionalized Freestanding TiO_2 Nanotube Arrays Coated with Ag Nanoparticles and Carbon Materials for Dye-Sensitized Solar Cells. *Appl. Sci.* **2017**, *7*, 576.
